# The Pitfalls of Calcitonin as a Tumor Marker: Real-Life Data of Patients with Elevated Basal Calcitonin Levels but Without Evidence of Medullary Thyroid Carcinoma

**DOI:** 10.3390/jcm15072500

**Published:** 2026-03-25

**Authors:** Ann-Kathrin Lederer, Constantin-Leonard Jacob Kessler, Nabila Bouzakri, Oana Lozan, Florian Wild, Katharina Theresa Rauschkolb-Olk, Heidi Rossmann, Hauke Lang, Thomas J. Musholt

**Affiliations:** 1 Section of Endocrine Surgery, Department of General, Visceral and Transplantation Surgery, University Medical Center Mainz, Johannes Gutenberg University Mainz, 55131 Mainz, Germany; ann-kathrin.lederer@uniklinik-ulm.de (A.-K.L.);; 2 Department of General and Visceral Surgery, Ulm University Hospital, 89081 Ulm, Germany; 3 Institute of Clinical Chemistry and Laboratory Medicine, University Medical Center Mainz, Johannes Gutenberg University Mainz, 55131 Mainz, Germany

**Keywords:** calcitonin, thyroid cancer, hormones, neoplasms, diagnosis, biomarkers, real-world data, hypercalcitoninemia, c-cell hyperplasia

## Abstract

**Background**: Calcitonin, a tumor marker primarily used to diagnose medullary thyroid carcinoma (MTC), can also be elevated in other conditions, complicating diagnosis. This study aims to provide a clinical evaluation of the real-world consequences of unexplained calcitonin elevation. **Methods**: We conducted a retrospective cohort study of patients with elevated basal calcitonin levels who presented at the Department of General, Visceral, and Transplantation Surgery, University Medical Center Mainz, between January 2015 and March 2025. Additionally, we reviewed electronic health records from 2007 onward for patients with ICD codes indicating calcitonin hypersecretion. Patients with confirmed MTC or genetic syndromes were excluded. **Results**: Of 345 patients with elevated calcitonin levels, 167 (48%) met the inclusion criteria, and 29 additional patients with calcitonin hypersecretion were identified via ICD, resulting in 167 patients analyzed. More than half of the patients were female (52%), had an average age of 53.9 years and a high prevalence of goiter (86%). Calcitonin levels were slightly elevated (<20 pg/mL) in 81% of cases and were above 50 pg/mL in only 10 patients. Surgery was performed in 77% of patients, mainly to exclude malignancy. Postoperatively, calcitonin normalized in 86% of patients but remained elevated in eight patients. Two of these patients were found to have false-positive results due to assay interference. Follow-up data were incomplete for a substantial proportion of patients, with a median follow-up of 4.6 months. The mortality rate was 4%, with causes unrelated to calcitonin levels. **Conclusions**: Elevated basal calcitonin levels, especially slightly elevated levels (<20 pg/mL), are common in clinical practice and often do not appear to be related to malignant disease, so careful investigation is required. Persistently elevated calcitonin levels justify further examinations, especially if other explanations can be ruled out. Only a few patients attend follow-up appointments, which makes patient follow-up challenging.

## 1. Introduction

About 2–5% of thyroid carcinomas are medullary thyroid carcinomas (MTCs), which makes them a rare but clinically significant form of thyroid carcinoma [1,2]. MTC most commonly occurs sporadically, accounting for approximately 50–75% of cases, or as part of a hereditary syndrome, e. g., the Multiple Endocrine Neoplasia (MEN) [3,4]. MEN is a rare autosomal-dominant inherited cancer syndrome that is divided into MEN1, MEN2 (formerly MEN2A), MEN3 (formerly MEN2B), MEN4 and MEN5 [5]. MTCs occur in MEN2 and MEN3, with patients with MEN3 in particular developing MTC in early childhood [6]. Recent research shows that the incidence rate, especially of sporadic MTC, is steadily increasing, which makes it necessary to have reliable early detection measures available [3,7]. The prognosis of MTC is generally less favorable than that of differentiated thyroid cancers due to its aggressive nature, potential for early metastasis and few effective drug therapies [8]. The only curative treatment for MTC is a radical surgical resection compromising total thyroidectomy and systematic lymph node dissection [2,9,10]. Early detection and diagnosis of MTC are crucial, as the clinical course and prognosis of this disease depend heavily on timely identification and intervention.

MTCs arise from a specialized neuroendocrine cell line, also known as C cells [8,11]. The main function of the C cells is the secretion of an amino peptide hormone called calcitonin [8,11]. The secretion of calcitonin reduces the amount of blood calcium in the human body [11,12]. It is therefore the functional antagonist of parathyroid hormone (PTH). Interestingly, in humans, calcitonin is not an essential hormone for life, unlike in fish and amphibians [13]. In fact, it is even debated whether calcitonin is an evolutionary remnant. If calcitonin secretion is impaired, such as after thyroidectomy, calcium homeostasis can still be effectively maintained [12,13,14]. Since calcitonin is only secreted by the C cells, the increase in calcitonin serves as an important diagnostic marker for MTC [1,8,15,16]. However, elevated basal serum levels of calcitonin can also occur after consuming calcium-rich food, nicotine or alcohol or in other pathological conditions such as hyperparathyroidism (HPT), chronic renal failure, gastritis or as a paraneoplastic syndrome in some neuroendocrine tumors (NET), which can complicate diagnostic accuracy and clinical interpretation [1,2,12,17,18,19]. Furthermore, the normal range of basal calcitonin varies according to age and gender, and can be false positive, e. g., with test-specific antibodies leading to an assay interference [1,8,12]. Up to a basal calcitonin value of 50 pg/mL in men and 30 pg/mL in women, the diagnosis of MTC is not certain, but the higher the value, the more likely it becomes [20]. A calcitonin level exceeding 100 pg/mL is considered diagnostic for MTC [16]. In recent years, several studies have attempted to further evaluate and validate the role of calcitonin in the diagnosis of MTC. Despite these efforts, the diagnostic accuracy of calcitonin in various clinical scenarios remains under-researched. Clinical guidelines are not fully consistent, with some recommending routine measurement of basal calcitonin, while others do not, reflecting ongoing uncertainty about its role in early detection [21]. Human anti-mouse antibodies (HAMA) may represent a potential source of false-positive results due to assay interference [1]. Although evidence for their effect on calcitonin assays is extremely limited, similar phenomena have occasionally been reported for related markers such as procalcitonin [22,23]. From our clinical experience as a high-volume thyroid surgery center, we recognize that there are situations in which serum calcitonin levels, even when above 100 pg/mL, can be misleading. The aim of this study was to systematically characterize patients with elevated basal serum calcitonin levels without histologically confirmed MTC in a real-world clinical setting. Rather than defining new diagnostic thresholds, we focused on identifying alternative causes for calcitonin elevation, evaluating the role of surgery, and outlining a clinically grounded approach to avoid unnecessary interventions in these patients. Even though calcitonin might be an excellent diagnostic tool in most patients, particular attention must be paid to patients in special clinical scenarios to avoid overtreatment and delayed diagnosis.

## 2. Materials and Methods

This study was a single-center retrospective cohort analysis that included all patients presenting to the Endocrine Surgery Section of the Department of General, Visceral and Transplant Surgery at the Mainz University Medical Center. The aim of the study was to investigate the real-life clinical characteristics of patients with elevated basal serum calcitonin levels in whom MTC had not been histologically confirmed. We hypothesized that a relevant proportion of patients with elevated basal serum calcitonin levels in routine clinical practice do not have histologically confirmed MTC and that markedly elevated values may, in rare cases, represent false-positive laboratory results. According to the regulations of the Ethics Committee of Rhineland-Palatinate, studies based exclusively on retrospective analysis of routinely collected clinical data do not require formal ethical approval. Nevertheless, all patients had previously provided informed consent for the use of their clinical data for research purposes.

### 2.1. Collection of Data

To identify eligible patients, we queried the electronic database of the Institute of Clinical Chemistry and Laboratory Medicine. All patients between 01/2015 and 03/2025, regardless of age and gender, and whose basal serum calcitonin was above the normal range were eligible for inclusion. In addition, to capture patients treated before 2015, we searched all electronic health records from 2007 onward for patients who were assigned to the ICD (International Statistical Classification of Diseases and Related Health Problems) for hypersecretion of calcitonin (E 07.0). Clinical data from patients who underwent surgery were obtained from our own entries in the EUROCRINE^®^ registry and from the institution’s electronic patient records. Patients who did not undergo surgery had data only in their electronic medical records, which were used for clinical analysis. Dates of death of the patients were obtained using the electronic patient files and by reading obituaries. Potential confounding factors known to influence calcitonin levels were extracted from the medical records whenever available. These included smoking status, excessive alcohol consumption, medication use (including calcium supplementation), vitamin D deficiency, renal insufficiency, thyroiditis, gastritis, hyperparathyroidism, pituitary disease, autoimmune diseases, and malignancies [1,2,12,17,18,19]. The decision to perform thyroid surgery was made according to standard clinical indications, including suspicious thyroid nodules on ultrasound, indeterminate or suspicious cytology, progressive nodule growth, compressive symptoms, or clinical suspicion of malignancy. Elevated calcitonin levels alone were generally not considered a sufficient indication for surgery.

### 2.2. Criteria of In- and Exclusion

Patients with the diagnosis of histopathologically confirmed MTC, MEN1/2 or related genetic syndromes were excluded from further evaluation. Patients without thyroid examination and duplicates were excluded; each patient was included only once. For patients with multiple calcitonin measurements, inclusion in the study was determined by the first documented elevated value, even if the patient had previous visits to our clinic.

### 2.3. Measurement of Calcitonin

In our department, all patients presenting for evaluation of thyroid nodules who do not have recent external laboratory results undergo calcitonin testing. Blood samples were taken on the day of the patients’ appointments. There was no planned fasting period before blood sampling. Blood samples were processed immediately. Basal serum calcitonin levels were measured using chemiluminescence immunoassays (CLIAs). Until 12 June 2019, measurements were performed using the Immulite 2000 system (Siemens, Forchheim, Germany) with Siemens calcitonin reagents, controls, and calibrators (normal range males: <8.4 pg/mL, females: <5 pg/mL). The assay measurement range was 2–2000 pg/mL, with higher values measured after dilution. From 12 June 2019 onwards, measurements were performed using the Liaison XL system (Diasorin S.p.A., Saluggia, Italy) with LIAISON^®^ Calcitonin II Gen reagents, control sets, and calibrators (normal range males: <11.8 pg/mL, females: <4.8 pg/mL. The measurement range was 1–2000 pg/mL, with higher values measured after dilution. To ensure comparability between assays, a correlation analysis was performed using 48 samples measured with both systems, resulting in a correlation coefficient of r = 0.95. All measurements were performed according to the manufacturer’s instructions by the Institute of Clinical Chemistry and Laboratory Medicine, University Medical Center Mainz. Both assays are also considered comparable in the literature [24].

### 2.4. Statistics

As it was unclear how many patients would show elevated basal calcitonin, it was not possible to calculate a sample size before study onset. This study is, therefore, an exploratory pilot trial. All data were entered into a predefined table and analyzed descriptively. Patients were divided into two groups. Group 1 included individuals who underwent thyroid surgery but whose histological reports did not confirm MTC. Group 2 consisted of patients who did not undergo surgery, either because MTC was not suspected based on clinical or imaging findings or because surgery was recommended due to suspected MTC but ultimately declined by the patient.

The analysis was carried out using SPSS (IBM^®^, Version 30). Numerical data were checked for normal distribution by the Kolmogorov–Smirnov test. Continuous numerical data are presented as mean and standard deviation or as median and range in case of non-normally distributed data. Categorical data are presented as the absolute number of patients with the specified characteristic and as a percentage of all included patients. To evaluate the influence of gender and age category (>55 vs. ≤55 years), chi-square tests were performed. The Wilcoxon rank-sum test was used to compare numerical data. The correlation analysis was carried out using Spearman’s Rho correlation. Missing data were not imputed. A *p*-value smaller than 0.05 was defined as significant.

## 3. Results

**Figure 1:**
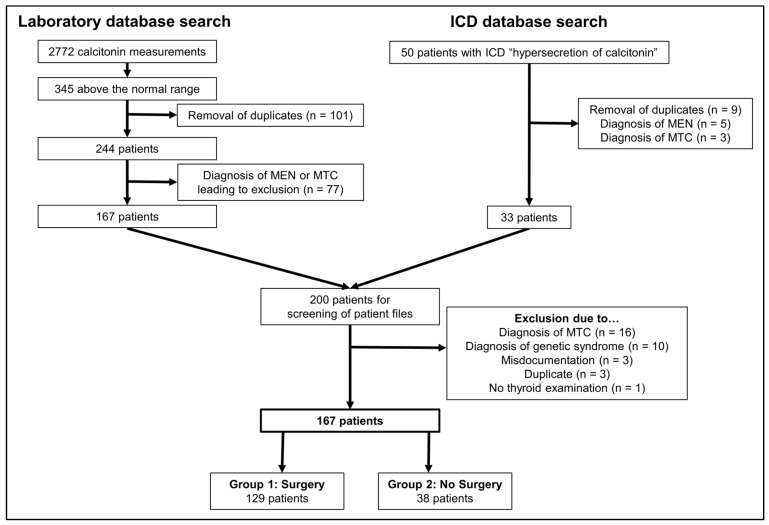
Process of patient selection.

In total, we analyzed the data of 167 patients with an elevated basal serum calcitonin level but without histopathological confirmed MTC. The whole process of screening and inclusion is shown in Figure 1.

Patients were identified through two sources: (1) calcitonin measurements in the laboratory database and (2) an ICD-based search for hypersecretion of calcitonin:

(1) Between 1 January 2015 and 2 October 2024, a total of 2772 calcitonin measurements were ordered in our outpatient clinic. Of these, 345 measurements were above normal. After excluding duplicates (some patients had more than one measurement), 244 patients remained with a calcitonin value above the normal range. Seventy-seven patients were excluded after a comparison with the EUROCRINE^®^ registry because they had MEN or MTC. Among the 244 patients with initially elevated calcitonin levels, 138 (57%) were not diagnosed with histologically confirmed MTC.
(2) The ICD search found 50 patients with a diagnosis of hypersecretion of calcitonin. Of these patients, nine cases were duplications, a further five had MEN and three had MTC, leaving a total of thirty-three patients for review of medical records.

Both datasets (patients identified via laboratory measurements and those identified through the ICD search) were merged, resulting in a total of 200 patients who underwent detailed medical record review. During this review, 34 patients were excluded due to misdocumentation (n = 3), duplicate records (n = 3) or diagnosis of genetic syndromes (n = 10), or MTC (n = 18). In addition, one patient with a benign cystic non-hormone-active pancreatic lesion had no thyroid examination, which is why we excluded the patient.

Characteristics of all patients are shown in Table 1. More than half of the patients were female (n = 86, 52%). The average age was 53.9 ± 15.6 years. One-hundred-seven patients (74%) originated from Germany. The second most common origin was Turkey (n = 6, 4%), followed by Russia (n = 3, 2%) and Syria (n = 3, 2%). A third of the patients suffered from hypertension (n = 52, 31%). Twenty-three patients (14%) suffered from renal insufficiency, ten of them in the final stage, so that dialysis was necessary. A goiter was diagnosed in 136 patients (86%). Table 1 summarizes also potential causes of hypercalcitoninemia, including thyroid and parathyroid disorders (goiter, thyroiditis, hyperparathyroidism) as well as renal insufficiency.

Results of ultrasound examination of the thyroid and parathyroid glands as described in the electronic patient file are shown in Table 2. Forty-seven patients (28%) suffered from HPT, but only ten patients were described as having parathyroid adenomas on ultrasound. In the total cohort, the median PTH level was 59.7 pg/mL (range 4.0 to 1906.6 pg/mL), and the median calcium level was 2.43 mmol/L (range 2.15 to 3.47 mmol/L). Data on alcohol consumption, vitamin intake, smoking status, and medication use were not available for all patients, so a comprehensive evaluation of these potential contributing factors could not be conducted.

### 3.1. Basal Calcitonin Levels

**Figure 2:**
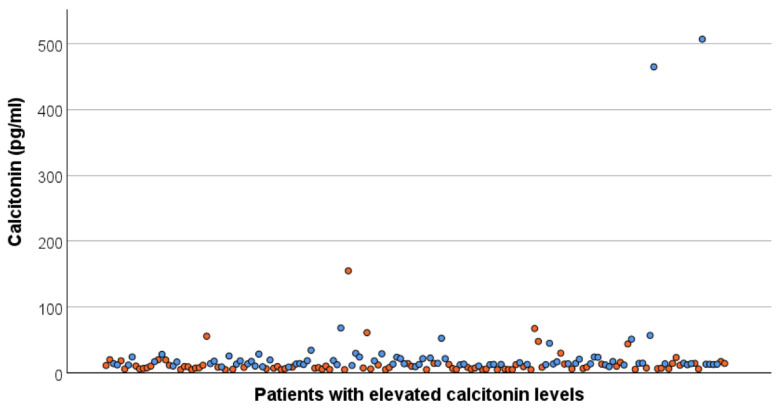
Initial basal serum calcitonin levels of all included patients. Female patients are indicated by an orange dot and male patients by a blue dot. The majority of patients (81%, n = 136) showed a slight increase in calcitonin (above normal reference range, but below 20 pg/mL).

The median initial basal serum calcitonin level of all patients was 12.6 pg/mL (range 4.8 to 507.0 pg/mL). A graphical representation of the values, separated by sex, is shown in Figure 2. In men, the median level was 14.0 pg/mL (range 8.6 to 507.0 pg/mL), while in women, it was 8.1 pg/mL (range 4.8 to 155.0 pg/mL). The majority of patients (81%, n = 136) showed a slight increase in calcitonin (above normal reference range, but below 20 pg/mL). Only ten patients had calcitonin levels above 50 pg/mL, three of whom had levels above 70 pg/mL. Significantly higher median preoperative calcitonin levels were measured in patients who had surgery compared to those without surgery (13.0 vs. 9.3 pg/mL, *p* = 0.024).

In the following, the results for the patients were analyzed separately according to surgical (Group 1) or conservative treatment (Group 2) and with postoperative measurement of calcitonin. More than a third of patients (n = 66, 40%) had a follow-up examination of calcitonin. An overview of calcitonin levels is shown in Table 3.

#### 3.1.1. Group 1: Basal Calcitonin Levels in Patients Who Underwent Surgery

Almost 80% of patients underwent surgery (n = 129, 77%). Patients who underwent surgery had an average age of 55.3 ± 14.8 years and were predominantly male (n = 72, 56%). In the operated male and female patients, the median preoperative basal serum calcitonin level was 14.1 pg/mL (range 8.6 to 465 pg/mL) and 8.5 pg/mL (range 4.8 to 155 pg/mL), respectively (Table 3). Surgery-related data such as indications and type of performed surgery are presented in Table 4.

Almost 80% of patients had a clinically or sonographically diagnosed goiter with nodular lesions of the thyroid gland. Sixty-five patients (50% of all operated patients) underwent thyroidectomy. In most of the patients (n = 47, 36% of all operated patients), the indication for surgery was the exclusion of a malignant tumor, mainly MTC, due to the results of calcitonin measurement and suspicious ultrasound findings. Twenty-eight patients (30%) underwent surgery for HPT (14 patients with primary and 14 patients with secondary HPT), including eight patients (7%) with a combination of HPT and symptomatic goiter. Of all surgically treated patients, 19 (15%) were histologically diagnosed with thyroid carcinoma, mainly papillary thyroid microcarcinomas, while 26 (20%) had thyroiditis.

Of all 129 patients who underwent surgery, postoperative basal serum calcitonin was measured in 59 patients (46%) (Table 3). These patients had an average age of 54.0 ± 14.2 years and were more frequently male (n = 37, 63%). The median preoperative basal serum calcitonin levels were 17.2 pg/mL (range 8.6 to 465 pg/mL) in male patients and 9.8 pg/mL (range 4.9 to 155 pg/mL) in female patients (Table 3). The median postoperative basal serum calcitonin levels were 0 pg/mL (range 0 to 433 pg/mL) in male patients and 2.1 pg/mL (range 0 to 161 pg/mL) in female patients (Table 3). Calcitonin normalized within one month after surgery in 86% (n = 51) of all patients with a postoperative calcitonin measurement. After thyroidectomy, calcitonin was below the detection limit in 37 of 39 patients (95%). There was no normalization of calcitonin after surgery in eight patients. All characteristics of these patients are shown in Table 5.

**Figure 3:**
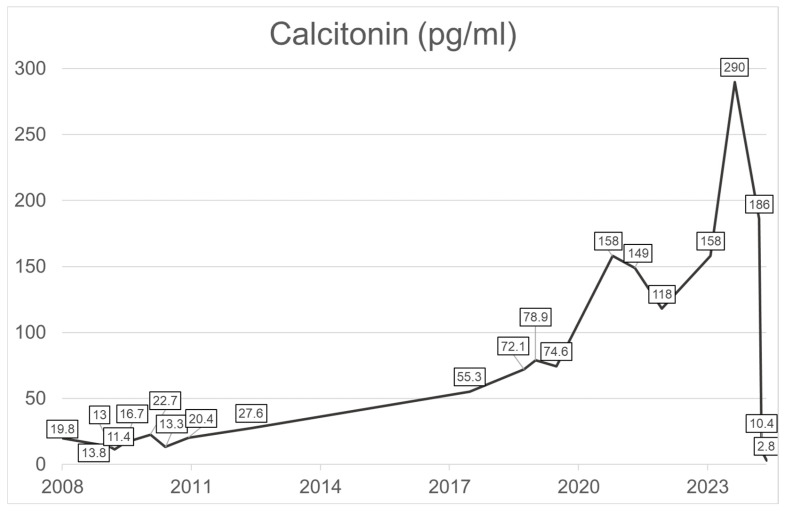
Basal serum calcitonin progression of patient 15 over more than 10 years. The patient underwent two surgeries (thyroidectomy 2009, lymphadenectomy 2024).

None of the patients without postoperative normalization suffered from HPT or renal insufficiency. Preoperative median calcitonin levels differed significantly between patients with and without postoperative normalization of calcitonin (14.0 vs. 25.7 pg/mL, *p* = 0.045). In patients 9, 31, 117 and 123, calcitonin decreased by more than half of the baseline value after surgery. Patient 66 had a postoperative increase in calcitonin. Three of the patients (patients 31, 66 and 148) were tested for HAMA, which remained negative for 31 and 148, but positive for 66. Patient 148 was tested with a different calcitonin assay due to suspected rheumatoid arthritis-related heterophile antibodies. The retest revealed normal results, emphasizing the presence of antibodies. Thus, in two patients, elevated calcitonin levels were confirmed to result from assay interference. Patient 31 suffered from thyroiditis but underwent thyroidectomy. Further two patients (123 and 117) had external reason for elevated calcitonin, as patient 123 took calcium continuously after surgery and patient 117 was taking a proton pump inhibitor. However, both had thyroidectomy, which is why it is questionable whether these causes can still lead to an increase in calcitonin levels postoperatively. Patient 86 suffered from NET, which can explain the elevated calcitonin levels [19]. Patient 15, who underwent thyroidectomy more than 15 years ago, had fluctuating calcitonin levels for over 10 years until they finally increased clearly (Figure 3). The first suspicion of occult MTC was expressed, but another five years passed before we saw the patient again. At that time, PET-CT revealed a cervical mass, which was identified as a suspicious lymph node by ultrasound, leading to the suspicion of a metastasis of occult MTC and indicating the need for lymphadenectomy. After surgery, the calcitonin levels normalized (2.4 pg/mL) and pathology confirmed lymph node metastasis of an occult MTC.

#### 3.1.2. Group 2: Basal Calcitonin Levels in Patients Without Surgery

Thirty-eight patients (23%) did not undergo surgery. Patients without surgery had an average age of 49.4 ± 17.4 years and were more frequently female (n = 29, 76%). Median initial basal serum calcitonin of male and female patients was 13.2 pg/mL (range 9.2 to 507.0 pg/mL) and 7.3 pg/mL (4.8 to 67.3 pg/mL), respectively (Table 3). The reasons for the calcitonin elevations remain largely speculative. Possible explanations for the elevated calcitonin levels included hyperparathyroidism (six patients, 16%), smoking (three patients, 8%), use of calcitonin-influencing medication such as proton pump inhibitors or antidepressants (three patients, 8%), younger age (three patients, 8%; including one patient with a complex non-thyroid genetic syndrome), as well as one case each of prolactinoma, autoimmune disease, gastritis, or vitamin-D deficiency (each representing 3% of patient). Thyroiditis was detected in laboratory tests in an additional eight patients (21%). One patient (3%) with a goiter not suspected of malignancy was tested for HAMA, which remained positive. Six patients had calcitonin levels above 20 pg/mL, with three of them having levels above 50 pg/mL. All patients with a calcitonin level between 20 and 50 pg/mL had the recommendation for surgery but refused. In one female patient, the cause of the calcitonin elevation remained unknown. Of the two male patients with levels above 20 pg/mL, one was a smoker with thyroiditis. The female patient was lost to follow-up, while both men showed no clinical signs of MTC at 4 and 12 months, respectively. Three patients had calcitonin levels above 50 pg/mL. Of the three patients with calcitonin levels above 50 pg/mL, two were diagnosed with bronchial carcinoma, while the third patient had a TIRADS V thyroid nodule highly suspicious for MTC. Surgery was recommended, but the patient refused and was lost to follow-up.

### 3.2. Correlation of Basal Calcitonin with Age and Thyroid- or Parathyroid-Related Markers

There was no correlation between the initial calcitonin levels and postoperative calcitonin levels, nor with age, thyroid-stimulating hormone (TSH), PTH, and calcium. We found a negative correlation between postoperative calcitonin levels and patients’ age (r = −0.273, *p* = 0.037) and TSH (r = −0.210, *p* = 0.006). There was a positive correlation between age and levels of PTH (r = 0.392, *p* < 0.001) and calcium (r = 0.287, *p* < 0.001). There was also a positive correlation between the calcium levels and levels of PTH (r = 0.319, *p* < 0.001). In the sex-specific analysis, the correlations of PTH and calcium with age were observed in female patients (PTH and age: r = 0.572, *p* < 0.001), calcium and age: r = 0.381, *p* < 0.001), whereas no correlations were found in male patients. In male patients, there were correlations between TSH and age (r = −0.238, *p* = 0.032). The correlation between postoperative calcitonin levels and age disappeared in the sex-specific analysis.

### 3.3. Influence of Age, Sex and Comorbidities on Calcitonin Levels of All Patients

For age analysis, patients were divided into two groups: those above (n = 87) and those below 55 years of age (n = 80). As expected, hyperparathyroidism was more frequently observed in the older patient group (39% vs. 16%, *p* = 0.001). Comorbidities such as arterial hypertension (44% vs. 18%, *p* = 0.001), diabetes mellitus (15% vs. 4%, *p* = 0.49), renal insufficiency (21% vs. 6%, *p* = 0.15), and coronary heart disease (14% vs. 3%, *p* = 0.31) were also more prevalent among older patients. There was no difference in the rate of surgery between younger and older patients (74% vs. 81%, *p* = 0.198). Calcitonin normalization after surgery was achieved equally in both groups. In the older group, a higher number of patients exhibited moderately elevated calcitonin levels (25% vs. 11%, *p* = 0.028). Statistical comparison of the absolute calcitonin values in age groups did not reach significance.

As expected, there was a clear difference in calcitonin levels between men and women (14.0 pg/mL vs. 8.1 pg/mL, *p* < 0.001). Arterial hypertension was more common among male patients (41% vs. 22%, *p* = 0.032), whereas psychiatric disorders were more prevalent in female patients (9% vs. 0%, *p* = 0.019). Women underwent surgical intervention less frequently (89% vs. 66%, *p* < 0.001) and were more likely to present with histologically thyroiditis (21% vs. 12%, *p* = 0.45). Calcitonin normalization was more frequently achieved in men than in women (43% vs. 19%, *p* < 0.001).

Basal calcitonin levels did not differ substantially between patients with and without goiter, with comparable median values (12.5 pg/mL vs. 12.8 pg/mL).

### 3.4. Clinical Follow-Up and Survival

Follow-up data were not available for 31 patients (19%), including one patient who died postoperatively. The median follow-up was 4.6 months (range 0 to 184 months) for all patients and 9.3 months (range 1 to 184 months) for patients (n = 136) who attended at least one follow-up examination. For 88 patients (53% of all patients), follow-up data were available for more than 6 months. Patients with surgery had a follow-up of 5.0 months (range 0 to 184 months), whereas patients without surgery had a median follow-up of 3.4 months (range 0 to 71 months). Only one patient was later diagnosed with (occult) MTC. None of the other patients with follow-up data showed clinical signs of MTC.

**Figure 4:**
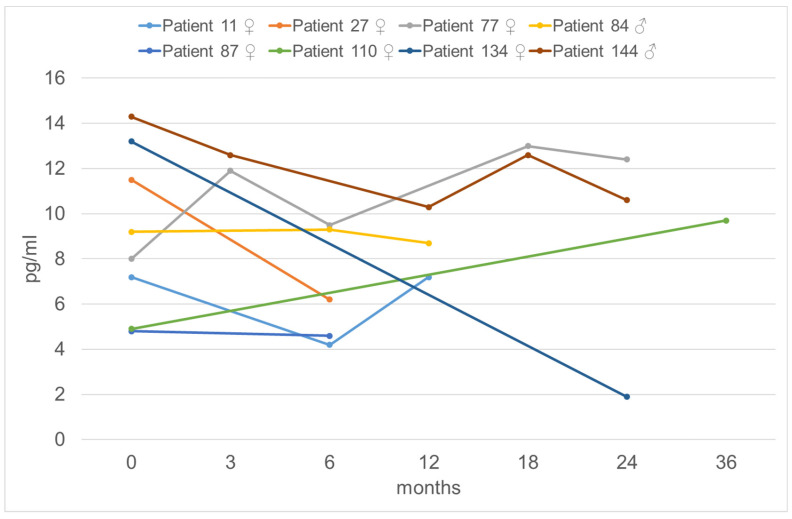
Basal serum calcitonin levels in patients (n = 8) who did not undergo surgery and who presented for calcitonin monitoring. The calcitonin level of patient 84 was measured with the Immulite assay (normal range males: <8.4 pg/mL, females: <5 pg/mL); all other patients were measured with the Liasion assay (normal range males: <11.8 pg/mL, females: < 4.8 pg/mL).

Of all patients without surgery, 14 (37%) presented to our clinic only once. A further 24 patients (63%) were seen again in our clinic, mainly for not-thyroid-related complaints. Only eight patients (21%) had a follow-up examination of calcitonin in our clinic. The course of calcitonin in these patients is shown in Figure 4.

Almost 4% of patients (n = 7) died. The median age at the time of death was 63 years (range 45 to 85 years). One patient died due to a progression of a poorly differentiated thyroid carcinoma. One patient died of a cerebral hemorrhage, three others of a non-thyroid-related cancer, and the cause of death is unknown in one patient. One elderly patient with multimorbidity died during the postoperative course due to decompensated heart failure.

## 4. Discussion

This study aimed to provide a structured clinical evaluation of patients with elevated calcitonin levels in the absence of histologically proven MTC. The primary intention was not to refine diagnostic thresholds but to better understand the real-world consequences of surgical and conservative management in these often ambiguous cases. Our data suggest that the combination of elevated calcitonin levels and suspicious thyroid nodules may frequently lead to surgical intervention. In contrast, mildly elevated calcitonin levels in otherwise unremarkable patients rarely result in surgery and, unfortunately, are often associated with incomplete or absent follow-up. Overall, 129 patients underwent surgery, of whom 77 had a complete resection of thyroid tissue, with no evidence of MTC on final histopathology. In the majority of these patients, only slight increases in calcitonin levels were observed, while the markedly elevated levels (>70 pg/mL) in two patients were confirmed to result from assay interference. In 86% of the operated patients, the calcitonin value returned to normal after surgery, indicating that the reason for the increase in calcitonin was mainly in the thyroid gland. This is not surprising, since the C cells that secrete calcitonin are found in the thyroid gland. In our cohort, only one patient (patient 15) was later diagnosed with occult MTC without known primary, which presented as a lymph node metastasis nearly 15 years after thyroidectomy. In general, in cases where no primary can be found, the possibility of ectopic tissue must be considered. Ectopic thyroid tissue is an exceptionally rare occurrence; however, the development of MTC within such tissue, while uncommon, cannot be entirely ruled out [25]. In our patient, no ectopic site of the primary MTC could be detected by positron emission tomography-computed tomography (PET-CT). Given the high cost and radiation exposure, PET-CT is reserved for patients with a strong suspicion of malignancy. In our cohort, most cases of calcitonin elevation could be attributed to causes other than MTC (known neuroendocrine tumor in patient 86, assay interference in patients 66 and 148, thyroiditis or medication in patients 31, 117 and 123). However, persistent calcitonin elevations after thyroidectomy remain unexplained. While prior thyroiditis or the use of calcitonin-influencing medications may have contributed before surgery, these factors should no longer play a role once the thyroid has been removed. It is therefore possible that the remaining elevations are due to ectopic calcitonin production from non-thyroidal sources. Kiriakopoulos et al. reported that calcitonin can be produced by tissues in the lungs, prostate, small intestine, thymus, liver, and parathyroid glands [14]. Additionally, malignant non-thyroidal tumors, such as lung cancer, can secrete calcitonin, as it was also seen in our study. In 2017, Uccella et al. provided an overview of pancreatic neuroendocrine tumors secreting calcitonin, indicating that only 11% of the examined tumors expressed calcitonin [26]. One patient reported to suffer from migraine. Interestingly, migraine is associated with a vasodilatory neuropeptide called calcitonin gene-related peptide (CGRP) [27,28]. Alternative RNA processing of the same gene as calcitonin leads to CGRP, but the regulation of synthesis is not fully understood [29]. It is well known that CGRP can be elevated in patients with migraine, even outside of acute attacks [30].

We found that 138 out of the initial 244 elevated calcitonin measurements (57%) were not associated with a histologically proven MTC. The majority of the patients had only slightly elevated calcitonin levels. Mild elevations of basal calcitonin levels are a well-recognized diagnostic challenge [31]. Costante et al. stated that the higher the calcitonin level, the higher the risk of MTC. Basal calcitonin levels above 100 pg/mL were always associated with the presence of MTC, but values between 10 and 100 pg/mL represent a diagnostic gray zone and are considerably more difficult to interpret [16]. The results of our study suggest that high calcitonin levels (>70 pg/mL) are indicative of either MTC, other malignant tumors such as lung cancer or diagnostic errors. Kwon et al. stated that a basal calcitonin level of 65 pg/mL is a suitable cut-off for diagnosing MTC [32]. Baykan et al. reported an even lower cut-off value of 46.5 pg/mL for the presence of MTC, which we cannot confirm with our data [20]. However, they also pointed out that this cut-off has a specificity of only 74%, meaning that some healthy individuals may be incorrectly classified as diseased. Results of the German StuDoQ Thyroid register support that mild calcitonin elevations are rare in MTC [33]. The consequences of overtreatment, such as unnecessary thyroidectomy, include the need for life-long thyroid hormone replacement and the risk of complications such as recurrent laryngeal nerve palsy, postoperative hemorrhage, or hypoparathyroidism. In our study, two patients with significantly elevated calcitonin underwent unnecessary thyroidectomy due to assay interference, despite having benign, asymptomatic goiter. Notably, a third of all of our patients underwent surgery due to suspected MTC, which was later not confirmed. The observations of our study illustrate how important it is to take calcitonin elevations seriously, but not to sharpen the knives immediately. Basal serum calcitonin levels should not be trusted blindly. Managing elevated calcitonin levels requires patience and careful judgment. Particularly for mild calcitonin elevations, reliable decision-making tools are lacking. Elevated calcitonin levels may occasionally result from analytical interference rather than true biological secretion. Known sources of assay interference include heterophile antibodies or human anti-mouse antibodies, which can lead to falsely elevated results [1,34]. In such cases, laboratory validation procedures are recommended [35]. These include repeat measurements using the same assay, confirmation using an alternative assay platform, and evaluation of dilution linearity to detect potential interference. Today, the clinical routine rarely involves the use of stimulation tests to differentiate elevated basal serum calcitonin levels. Patients with MTC show a significantly greater increase in calcitonin after intravenous administration of pentagastrin or calcium compared to those without MTC [36,37,38]. The pentagastrin test was frequently used in the past, but is no longer available in Germany due to its unfavorable side effects, including, for example, nausea, flushing and headache [14,38]. As an alternative, the calcium stimulation test has been discussed, but it has not yet become a standard part of clinical practice also due to side-effects [21,38]. According to the ATA guideline, provocation testing can be performed, whereas the recently published German guideline recommends caution: due to poorly defined cut-off values, limited differentiation, and potential side effects, routine stimulation tests are generally discouraged [1,21]. In the last decade, there has been much discussion about whether ultrasound-guided fine-needle aspiration cytology (FNAC) and the measurement of calcitonin in the aspirate can contribute to the diagnosis. FNAC alone for the diagnosis of MTC is equivalent to a coin flip in terms of diagnostic accuracy, as a systematic review showed [39]. Supplementing FNAC by testing the aspirate for calcitonin leads to better diagnostic accuracy [40,41]. In addition to FNAC and calcitonin measurement in the aspirate, other adjunctive diagnostic tools, such as FNAC washout assays, have been discussed in the literature, but they have not become widely adopted to date and are not recommended in the current German guidelines [21]. However, it is not always possible to puncture suspicious nodules due to their size and location. Furthermore, FNAC is not ideal for nodules suspicious for MTC, as desmoplasia, an important prognostic marker in MTC, can be inadequately assessed following FNAC [42]. Differentiating between MTC and non-MTC in patients with only mildly to moderately elevated calcitonin levels remains difficult. Even mildly elevated tumor markers can cause an enormous psychological burden for patients, leading to anxiety, and it is the responsibility of physicians to guide them through the complexities of calcitonin evaluation, explaining both its utility and limitations. Calcitonin remains a valuable tool for diagnosing and monitoring MTC, but its results must always be interpreted in the context of the clinical presentation. The consideration of the clinical context is consistent with the 2015 guidelines on MTC, which recommend further imaging only when post-thyroidectomy basal serum calcitonin levels exceed 150 pg/mL [1]. Unnecessary procedures can contribute to increased healthcare costs, representing an additional burden for both patients and the healthcare system. However, our findings underscore the importance of guideline-recommended monitoring in patients with elevated calcitonin levels, while also illustrating the difficulties inherent in ensuring consistent and comprehensive follow-up [1]. The increase in calcitonin can be a trivial matter, but it can also be an early sign of MTC, sometimes also of metastasized MTC without known primary, as seen in our cohort. It is important that surgeons also pay attention to slightly elevated calcitonin levels and provide colleagues with recommendations for further treatment. In case of doubt, an experienced center should always be contacted to ensure the best possible treatment for the patient.

### Limitations

Our study is a retrospective analysis, which is always subject to potential documentation errors. A further limitation of this study is the absence of a comparison cohort, such as patients with normal calcitonin levels or patients with confirmed MTC. Consequently, our data do not allow the definition of diagnostic thresholds but rather provide a descriptive characterization of patients with elevated calcitonin levels in whom MTC was not confirmed. In our cohort, several causes of hypercalcitoninemia, such as goiter, thyroiditis, hyperparathyroidism, and renal insufficiency, were systematically assessed. However, information on smoking and medication use could not be reliably obtained for all patients, particularly those who were not surgically treated. Consequently, it was not possible to control for all potential confounding variables in the analysis. Furthermore, it cannot be ruled out that patients ate immediately before the blood sample was taken, as it was not documented whether patients were fasting at the time of blood sampling. It is known that basal serum calcitonin levels are affected by food intake [43]. A substantial proportion of our patients did not participate in follow-up evaluations, leaving uncertainty as to whether some may have developed MTC over time. The median follow-up time in our cohort was relatively short, which limits the ability to assess long-term outcomes of patients with elevated calcitonin levels. Given the typically slow tumor biology of MTC, longer observation periods would be required to reliably evaluate the long-term risk of malignancy and the clinical significance of mild calcitonin elevation. Our findings should primarily be interpreted as a descriptive characterization of patients with elevated calcitonin levels in a real-world clinical setting rather than as an assessment of long-term oncologic outcomes. Our cohort is inherently selected, as it originates from a surgical department, and we only see patients considered to have potential surgical indications. Nonetheless, our cohort of 167 patients represents a moderately sized group, allowing a meaningful evaluation of clinical outcomes.

## 5. Conclusions

In an era of increasing biomarker-driven diagnostics, this study highlights the importance of considering the clinical context when interpreting elevated calcitonin levels. Elevated calcitonin levels without histological proven presence of MTC are not uncommon in the clinical routine of an endocrine department. Calcitonin levels without confirmed MTC can be associated with diagnostic errors or other malignant tumors, which should be kept in mind when clinical findings and calcitonin levels do not align. Despite this, the possibility of occult MTC should not be entirely ruled out, even though it is extremely rare. In our cohort, a considerable proportion of patients with elevated calcitonin levels underwent surgery, underscoring the potential consequences of misinterpretation and the importance of careful clinical evaluation. These observations suggest that mildly elevated basal calcitonin levels should be interpreted with caution, as they may be associated with benign conditions that do not necessarily require aggressive interventions such as surgery. In cases of diagnostic uncertainty, repeated testing, the use of alternative assays, or interdisciplinary consultation with experienced endocrine centers may be helpful to support clinical decision-making. The findings of this study should be interpreted in light of the retrospective design and the limited follow-up of the cohort.

## Figures and Tables

**Table 1:** Sociodemographic and baseline medical characteristics of patients. Documentation was not available for all parameters for all patients; the percentages refer to all documented results. Data are presented as mean ± standard deviation, n (percentage of patients), or median (range), depending on the type and distribution of the variables.

Parameter	Results
Age (years)	53.9 ± 15.6
Sex (male/female)	81/86 (48/52%)
Origin (Germany/other)	107/37 (74/26%)
TSH (mU/L)	1.0 (0.01 to 11.2)
Parathyroid hormone (pg/mL)	59.7 (4.0 to 1906.6)
Calcium (mmol/L)	2.43 (2.15 to 3.47)
Previous surgery *	34/129 (21/79%)
Pre-existing diseases (yes/no)	Sorted by frequency
- Hypertension	52/111 (32/68%)
- Renal insufficiency	23/143 (14/86%)
- Diabetes mellitus	16/147 (10/90%)
- Cancer	16/147 (10/90%)
- Orthopedic disease	15/148 (9/91%)
- Coronary heart disease	14/149 (9/91%)
- Autoimmune disorder	13/150 (8/92%)
- Psychiatric disorders	8/155 (5/95%)
- Infectious disease	8/155 (5/95%)
- Gastritis or GERD	7/156 (4/96%)
- Pulmonary disease	7/156 (4/96%)
- Other atherosclerotic diseases	7/156 (4/96%)
- Neurological disease	6/157 (4/96%)
- Atrial fibrillation	6/157 (4/96%)
- Adrenal disease	4/159 (3/97%)
- Diseases of the pituitary gland	4/159 (3/97%)
- Heart failure	4/159 (3/97%)
Goiter (yes/no)	136/29 (82/18%)
Hyperthyroidism (yes/no)	26/141 (16/84%)
Autonomous nodules (yes/no)	18/147 (11/89%)
Hyperparathyroidism (yes/no)	47/120 (28/72%)

* all types of surgery, not only those related to the neck; GERD = gastroesophageal reflux disease; TSH = thyroid-stimulating hormone.

**Table 2:** Results of ultrasound examination of the thyroid and parathyroid glands as described in the electronic patient file. In case of missing TIRADS (Thyroid Imaging Reporting and Data System) classification, descriptions that could be classified according to TIRADS were assigned to the classification. If no classification could be assigned, the characteristics of the nodule are reported.

Results	n (%)
Nodule(s) *	38 (23%)
Hypoechoic nodule(s)	14 (8%)
Cystic nodule(s)	7 (4%)
Complex nodule(s)	6 (4%)
Thyroid gland larger than normal	4 (3%)
TIRADS 2	1 (1%)
TIRADS 2–3	5 (3%)
TIRADS 3	6 (3%)
TIRADS 3–4	5 (3%)
TIRADS 4	8 (5%)
TIRADS 4–5	8 (5%)
TIRADS 5	7 (4%)
Parathyroid adenoma	10 (6%)
Without pathologic findings	12 (7%)
Without documentation	35 (21%)

* Description was only “nodularly altered or nodule” without any further specification.

**Table 3:** Basal serum calcitonin levels in the overall cohort (n = 167) and in subgroups of patients with surgery (n = 129), surgery with follow-up (n = 59), and without surgery (n = 38). Values are presented as median (range). The table also summarizes postoperative normalization rates and potential alternative causes of elevated calcitonin levels in non-operated patients.

**Initial basal serum calcitonin level (n = 167)**		12.6 pg/mL (range 4.8 to 507.0 pg/mL)
*Male (n = 81)*		*Female (n = 86)*
14.0 pg/mL (range 8.6 to 507.0 pg/mL)		8.1 pg/mL (range 4.8 to 155.0 pg/mL)
*Category*		*Patients n (% of all patients)*
<20 pg/mL		136 (81%)
20–50 pg/mL		21 (13%)
50–70 pg/mL		7 (4%)
>70 pg/mL		3 (2%)
**Basal serum calcitonin levels in patients, who underwent surgery (n = 129)**		
*Male (n = 72)*		*Female (n = 57)*
14.1 pg/mL (range 8.6 to 465 pg/mL)		8.5 pg/mL (range 4.8 to 155 pg/mL)
**Basal serum calcitonin levels in patients, who underwent surgery with follow-up (n = 59)**		
*Male (n = 37)*		*Female (n = 22)*
*Pre*	17.2 pg/mL (range 8.6 to 465 pg/mL)	9.8 pg/mL (range 4.9 to 155 pg/mL)
*Post*	0 pg/mL (range 0 to 433 pg/mL)	2.1 pg/mL (range 0 to 161 pg/mL)
Normalization after surgery		51 (86%)
Persistent elevation		8 (14%)
Persistent after thyroidectomy		2 (4%)
**Basal serum calcitonin levels in patients without surgery (n = 38)**		
*Male (n = 9)*		*Female (n = 29)*
13.2 pg/mL (range 9.2 to 507.0 pg/mL)		7.3 pg/mL (4.8 to 67.3 pg/mL)
Possible explanations for calcitonin levels		Patients n (%)
- thyroiditis		8 (21%)
- hyperparathyroidism		6 (16%)
- smoking		3 (8%)
- use of calcitonin-influencing medication		3 (8%)
- younger age		3 (8%)
- prolactinoma		1 (3%)
- autoimmune disease		1 (3%)
- gastritis		1 (3%)
- vitamin-D deficiency		1 (3%)
- human anti-mouse antibodies (HAMA)		1 (3%)
- unknown		10 (26%)
<20 pg/mL		32 (84%)
20–50 pg/mL		3 (8%)
>50		3 (8%)

**Table 4:** Type of surgery, indications leading to surgery and histopathological results; percentage of all patients.

Type of Surgery	n (%)
Thyroidectomy	65 (39%)
Parathyroidectomy	25 (15%)
Thyroidectomy + Parathyroidectomy	8 (5%)
Left hemithyroidectomy	9 (5%)
Right hemithyroidectomy	6 (4%)
Left hemithyroidectomy + Parathyroidectomy	1 (1%)
Right hemithyroidectomy + Parathyroidectomy	2 (1%)
Thyroidectomy of remaining tissue	4 (2%)
Other *	9 (5%)
No surgery	38 (23%)
**Indication for Surgery**	
Exclusion of malignant tumor	47 (28%)
Hyperparathyroidism	29 (18%)
Symptomatic goiter	22 (13%)
Uncontrolled hyperthyroidism or autonomous nodules	12 (7%)
Hyperparathyroidism + symptomatic goiter	9 (5%)
Confirmed malignant tumor (non-MTC)	10 (6%)
No surgery	38 (23%)
**Histopathological Results ^+^**	
Malignancy	19 (15%)
C cell hyperplasia	23 (18%)
Thyroiditis	28 (22%)

* Thyroid isthmusectomy (with and without parathyroidectomy or in combination with left hemithyroidectomy), resection of local tumor recurrence, exploration without resection, resection of ectopic thyroid tissue, resection of the small gut, mesenteric lymphadenectomy and nodule enucleation; + percentage of all patients who underwent surgery; MTC = medullary thyroid carcinoma.

**Table 5:** Characteristics of patients without normalization of basal serum calcitonin after surgery.

ID	Age (Years)	Sex	Calcitonin		TSH (mU/L)	Ultra-Sound Results	Goiter	Surgery	Preexisting Diseases	Malignancy	Histology	(Possible) Cause of Calcitonin
			Before (pg/mL)	After (pg/mL)								
9	49	f	24.0	10.3	0.85	TIRADS 4a	yes	left hemithyroidectomy	migraine	no	goiter	unknown *
15	50	f	19.8	13.8	0.76	n/a	yes	thyroidectomy	none	no	thyroiditis and C cell hyperplasia	occult MTC
31	30	f	29.0	8.5	1.05	complex	yes	thyroidectomy	none	yes	thyroiditis and microPTC	thyroiditis *
66	16	f	155.0	161.0	0.91	complex	yes	thyroidectomy	none	no	thyroiditis	HAMA
86	60	m	21.6	19.9	2.4	n/a	no	mesenteric lymphadenectomy	neuroendocrine tumor (NET) of the small gut	yes	lymph node metastasis of NET	NET
117	35	f	47.7	15.5	1.92	nodule	yes	thyroidectomy	GERD and gastritis	no	goiter	proton pump inhibitor *
123	60	f	29.8	12.8	1.41	hypoechoic	yes	thyroidectomy	gastritis	yes	PTC	calcium intake *
148	64	m	465	433	0.7	complex	yes	thyroidectomy	coronary heart disease, rheumatoid arthritis	no	goiter	heterophile antibodies

f = female, m = male, n/a = not available due to missing documentation, GERD = gastroesophageal reflux disease, NET = neuroendocrine tumor, PTC = papillary thyroid carcinoma, HAMA = human anti-mouse antibodies; * patient 9 reported to be a non-smoker, had no abuse of alcohol and no relevant medication intake; due to thyroidectomy in patients 31,117 and 123, it must be questioned whether these reasons can still lead to an increase in calcitonin postoperatively. Normal range for calcitonin of patients 9, 15, 31: <4.8 pg/mL; normal range for calcitonin of patients 86, 156: <11.8 pg/mL (Liasion assay); normal range for calcitonin of patients 117, 123: <5 pg/mL; normal range for calcitonin of patient 148: <8.4 pg/mL (Immulite assay).

## Data Availability

The data presented in this study are available on reasonable request from the corresponding author.

## Abbreviations

The following abbreviations are used in this manuscript:

CGRP	calcitonin gene-related peptide
CLIA	chemiluminescence immunoassays
GERD	gastroesophageal reflux disease
HAMA	human anti-mouse antibodies
HPT	hyperparathyroidism
ICD	international statistical classification of diseases and related health problems
MEN1	multiple endocrine neoplasia type 1
MEN2	multiple endocrine neoplasia type 2
MTC	medullary thyroid carcinoma
n/a:	not available due to missing documentation
PTC	papillary thyroid carcinoma
PTH	parathyroid hormone
TIRADS	Thyroid Imaging Reporting and Data System
TSH	thyroid-stimulating hormone
